# Balneotherapeutic effects of high mineral spring water on the atopic dermatitis-like inflammation in hairless mice via immunomodulation and redox balance

**DOI:** 10.1186/s12906-017-1985-8

**Published:** 2017-10-13

**Authors:** Johny Bajgai, Ailyn Fadriquela, Jesmin Ara, Rahima Begum, Md Faruk Ahmed, Cheol-Su Kim, Soo-Ki Kim, Kwang-Yong Shim, Kyu-Jae Lee

**Affiliations:** 1 Department of Environmental Medical Biology, Wonju College of Medicine, Yonsei University, Wonju, Gangwon 26426 Republic of Korea; 2 Department of Global Medical Science, Wonju College of Medicine, Yonsei University, Wonju, Gangwon 26426 Republic of Korea; 3 Department of Microbiology, Wonju College of Medicine, Yonsei University, Wonju, Gangwon 26426 Republic of Korea; 4 Department of Internal Medicine, Wonju College of Medicine, Yonsei University, Wonju, Gangwon 26426 Republic of Korea; 5 Institute for Poverty Alleviation and International Development (IPAID), Yonsei University, Wonju Campus, Wonju, Gangwon 26493 Republic of Korea

**Keywords:** Atopic dermatitis, Balneotherapy, High mineral spring water, Immunomodulation, Redox balance

## Abstract

**Background:**

Atopic dermatitis (AD) is a chronic relapsing allergic inflammatory skin disease that currently affects millions of children and adults worldwide. Drugs used to treat these inflammatory diseases include anti-histamines, corticosteroids and calcineurin inhibitors but these drugs have their limitations such as adverse effects with their long-term usage. Thus, researcher’s interest in several alternative and complementary therapies are continually growing and balneotherapy is one of these approaches. Therefore, we investigate the bathing effect of high concentration mineral spring water (HMW) on redox balance and immune modulation in 2,4-dinitrochlorobenzene (DNCB)-induced atopic dermatitis like inflammation in hairless mice.

**Methods:**

We induced AD-like inflammation by application of DNCB on the dorsal skin of female skh-1 hairless mice. The mice were treated with 100% pure HMW (PHMW) and 10% diluted HMW (DHMW) through bathing once a day for 4 weeks. Tacrolimus ointment (0.1%) was used as positive control (PC) and only DNCB treatment as negative control (NeC) group. The severity of skin lesion inflammation was assessed through clinical scoring and observing scratching behavior. Levels of immunoglobulin E (IgE) and inflammatory cytokines in serum were detected by ELISA and multiplex bead array system, and the levels of oxidative stress-related biomarkers and antioxidant enzyme were also measured.

**Results:**

We found that HMW significantly decreased the scratching behavior in PHMW and DHMW groups at the 2nd week and in PHMW group at 4th week compared to NeC group. Likewise, serum IgE level was significantly decreased in DHMW group as compared to NeC group. In line, the level of inflammatory cytokines in serum such as interleukin (IL)-1β, IL-13 and tumor necrosis factor-α were significantly inhibited in PHMW and DHMW groups compared to NeC group. In parallel, total reactive oxygen species (ROS) of serum level was significantly decreased in PHMW treatment groups compared to NeC group. Consistently, serum malondialdehyde (MDA) level in PHMW group was lower than in NeC group. By contrast, glutathione peroxidase (GPx) activity was significantly enhanced in PHMW than NeC.

**Conclusion:**

Collectively, our study indicates a balneotherapeutic effect of HMW on DNCB-induced AD like inflammation in hairless mice via immunomodulation and redox balance.

## Background

Atopic dermatitis (AD) is a chronic relapsing allergic inflammatory skin disease, and currently affects millions of children and adults worldwide with its prevalence increasing two to three times over the past three decades [1, 2]. Although the exact etiology of AD has not been completely elucidated, a variety of causal factors including environmental, psychological, pharmacological [3], immunological and genetic [4] have been reported. In the therapeutic point of view, AD is featured by an impairment of the skin-barrier function, increased oxidative stress, dysfunctional immune system and elevated serum immunoglobulin E (IgE) levels [1, 3]. To alleviate these pathognomic feature, conventional drugs like anti-histamines, corticosteroids, and calcineurin inhibitors have been used to treat these inflammatory allergic disorders. However, these drugs have their own limitations, such as the short term usage (2-4 weeks), which is insufficient for clinical effectiveness, adverse side effects, and intolerance. For instance, long-term usage of these drugs is known to suppress the hypothalamic-pituitary-adrenal axis and related sequelae [5, 6]. To overcome this, clinicians’ interests in the alternative and complementary therapies are continually growing. Balneotherapy is one of these candidates to ameliorate AD with or without conventional medication.

Bathing in spring water (balneotherapy) has been widely used as a therapeutic tool for the treatment of skin diseases like acne, AD and psoriasis, and is prescribed by some European countries around the world [7–9]. Balneotherapy uses mineral water that is originates from different natural springs, and according to their location may be low mineralized (0.6-2 mg/L), mildly mineralized (>2-10 mg/L) or highly mineralized (>10 mg/L) water [10, 11]. Cumulative studies have hinted that the application of minerals like sulphur [12], manganese [13], magnesium [14], zinc [15], selenium and strontium [16] might exert gross therapeutic effects on skin diseases in human and DNCB-induced AD like inflammation in hairless mice. Another mechanistic evidence suggested that body exposure to mineral water at the spa would beneficially affect the immune system and antioxidant mechanism [17, 18]. Of these, a report in Japan showed the therapeutic effect of balneotherapy on AD [18]. Apart from this, the moderate level of some minerals such as manganese and sulfur in thermal spring water have been reported to be bactericidal against *Staphylococcus aureus* (SA) commensally resident in AD patient’s skin [13, 19]. Despite of these intermittent and superficial evidence of balneotherapy against AD, balneotherapy with higher levels of complex minerals (magnesium, calcium, chlorine, manganese, sulphur and strontium) in spring water is poorly documented, and further unclear about the detailed mechanisms of immunomodulation and redox balance in AD like inflammation. To address this issue, using natural mineral spring water, which is known for higher levels of complex minerals (Table 1), we investigated the balneotherapeutic effects on immunomodulation and redox balance in 2,4-dinitrochlorobenzene (DNCB)-induced atopic dermatitis like inflammation in hairless mice.

**Table 1:** Hydrochemical analysis of high mineral spring water

Mineral Content	Standard (mg/L)	HMW (mg/L)
Potassium (K^+^)	5	9.84
Magnesium (Mg^2+^)	25	183
Calcium (Ca^2+^)	5-500	2820
Sodium (Na^+^)	200	2900
Chlorine (Cl^−^)	250	9660
Sulphate (SO_4_^2−^)	250	1000
Lithium (Li)	0.02	14.1
Strontium (Sr)	0.46	91.6
Manganese (Mn)	0.3	21.0
Lead (Pb)	0.05	0.05
Zinc (Zn)	1.0	0.03
silicon dioxide (SiO_2_)	1-30	11.8
Iron (Fe)	0.3	0.02
Copper (Cu)	0.003	0.03
Fluoride (F^−^)	1.5	0.52
pH		7.53
Total dissolved solids	500	17,766

The above analysis is performed by using the inductively coupled Plasma-Mass Spectrometer (ICP-MS) and a Thermo Scientific iCAP 6500 duo Inductively Coupled Plasma-Atomic Emission Spectrometer (ICP-AES) by Korea Institute of Geosciences and Mineral Resources (Daejeon, Republic of Korea). *HMW* High mineral spring water

## Methods

### Experimental animals

Five-week-old female SKH-1 hairless mice with the mean weight (mean ± SD) 25 ± 4.2 g were purchased from Orient Bio Inc. (Seongnam, Republic of Korea) and used in carrying out the studies. The mice were obtained at the small unit of animal care and use department in Wonju College of Medicine, Yonsei University, Republic of Korea.

### Housing and husbandry

Handling of mice was done in accordance with the use and care protocols of Institutional Animal Care and Committee (IACUC) at Wonju College of Medicine, Yonsei University, Republic of Korea. The mice were kept in spacious plastic cages (390 × 275 × 175 mm) with wood shaving bedding and identified by labeling with surgical skin markers marking at the tail. They were acclimatized for 7 days to the housing environment prior to treatment and were maintained in a controlled environment with atemperature of 22 ± *2* °C and 40-60% humidity under a 12:12-h light-dark cycle. Standard rodent chow food (5 L79, PMI Nutrition®, LAND O’LAKES, INC, Minnesota, USA) and primary filtered water were supplied free to access until the end of the experiment. At the start of the experiment, 50 mice were randomized into five groups, five mice each cage (*n* = 10 respectively) as follows: Normal control group (NC), Negative control group (NeC) treated with DNCB only + DW bathing, Positive control group (PC) treated with DNCB +0.1% tacrolimus ointment + DW bathing, 100% pure high concentration mineral water (PHMW) group treated with DNCB+ PHMW bathing, and 10% diluted high concentration mineral water (DHMW) group treated with DNCB + DHMW bathing. The study protocol of the experiment was approved by the Institutional Animal Care and Use Committee (IACUC) at Wonju campus, (Ethical approval no: YWC-160513-1) Yonsei University, Gangwon, Wonju, and Republic of Korea. All the experiments were conducted between 7 a.m. and 6 p.m. to minimize the effects of environmental changes.

### Preparation of experimental water

A colorless clear solution of natural high mineral spring water (HMW) was supplied from Tae chang Co.Ltd. (Gyeokpo, Buan-gun, Republic of Korea). Mineral compositions of HMW were analyzed by a Thermo Electron x Series Inductively Coupled Plasma-Mass Spectrometer (ICP-MS) and a Thermo Scientific iCAP 6500 duo Inductively Coupled Plasma-Atomic Emission Spectrometer (ICP-AES) in Korea Institute of Geosciences and Mineral Resources (Daejeon, Republic of Korea) and the result was as Table 1. All the experimental water were stored in the big plastic container covered with a lid at 4 °C to protect from light and humidity until use. DHMW was prepared by 10% dilution of PHMW. For the treatment of NeC and PC groups, distilled water (DW) was used.

### Induction of allergic dermatitis with DNCB in skh-1 hairless mice

**Fig. 1:**
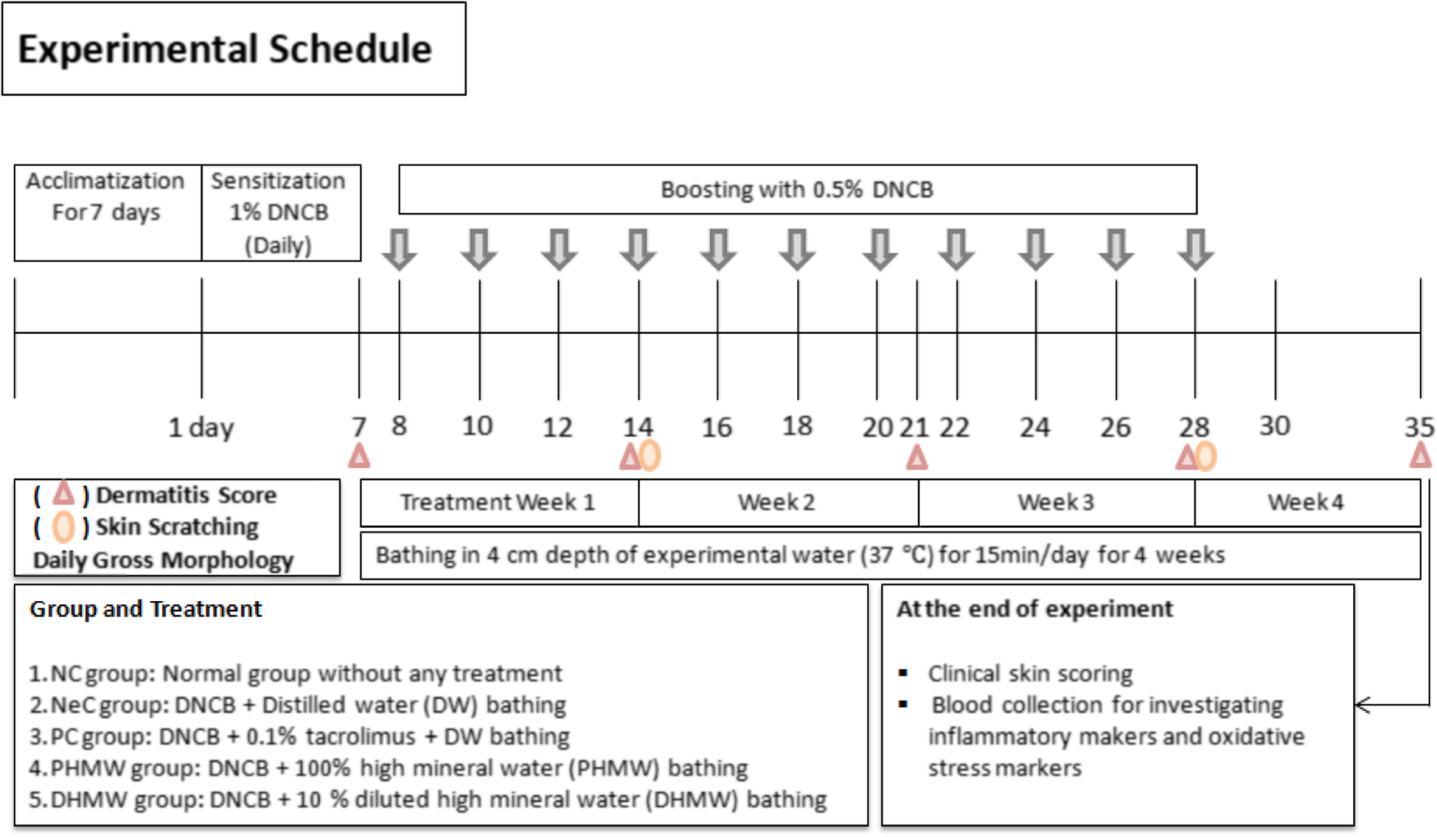
Scheme of the experimental procedure. To induce AD-like inflammation 1% DNCB was topically applied to the dorsal back skin of female skh-1 hairless mice for 1 week once a day and boosted with 0.5% DNCB every alternate day for 3 weeks. After 3 weeks of boosting and bathing treatment, one more week of only bathing treatment was followed. Mice were either treated with Negative control group (NeC): only DNCB+ DW bathing, Positive control group (PC): DNCB +0.1% tacrolimus ointment + DW bathing; 100% pure high concentration mineral water (PHMW) group (PHMW): DNCB + PHMW bathing; 10% diluted high concentration mineral water (DHMW) group (DHMW): DNCB + DHMW bathing. On day 35, mice were sacrificed and blood samples were collected for further analysis

AD-like inflammatory skin lesions were induced in skh-1 hairless mice by sensitization with 200 μL/mouse/day 1% DNCB (dissolved in a 3:1 mixture of acetone and olive oil) for 1 week, and boosted with 150 μL/mouse of 0.5% DNCB every alternate day for 3 weeks according to previous established methods [20]. The 3 weeks of boosting and bathing was followed by one more week of bathing with sample waters only. DNCB solutions were topically applied to dorsal skin (approximately 4 cm^2^) on each mouse except NC mice. In intact PC mice, tacrolimus ointment (0.1% ProtopicCo.Ltd. Osaka, Japan) was topically applied on the dorsal skin, seven times a week for 4 weeks (day 8-35). After a total of 4 weeks treatment, mice were anesthetized with isoflurane (Hana Pharm. Co., Hwaseong, Republic of Korea) in the mixture of 70% N_2_O and 28.5% O_2_ to minimize suffering and distress and blood samples of all the mice were collected from retro-orbital veins in EDTA vacutainer tubes and kept in ice packs. Immediately after blood collection, mice were sacrificed by cervical dislocation. The collected blood sample was centrifuged for 5 min at 14000 rpm and the separated serum was stored in −80 °C before use. A time line diagram for this experiment is shown in (Fig. 1).

### Bathing method for treatment after induction of AD-like inflammation

Five mice were freely bathed in a plastic cage (390 × 275 × 175 mm) containing 4 cm depth of PHMW, DHMW and DW respectively for 15 min/day for 4 weeks. The mice of both PHMW and DHMW groups were bathed in PHMW and DHMW, and PC and DNCB control group were bathed in DW to provide the same bathing condition. All the experimental water were warmed around 37 °C before bathing.

### Evaluation of the skin severity

The dermatitis severity was assessed by using skin scoring procedure, the frequency of scratching and skin test after triggering AD via DNCB. The dermatitis skin scoring procedure assessed eczema area and a severity index scoring system applied as follows: 0, no symptoms; 1, mild symptom; 2, moderate symptom; 3, severe symptom. The overall dermatitis score was defined as the sum of scores for erythema, edema, excoriation and scaling/dryness. The skin scoring was assessed once a week during the 4 weeks of treatment. Simultaneously, scratching actions such as rubbing their dorsal skin with their hind paws, their nose and ears were counted on 2nd and 4th week within 15 min in triplicate observation.

### Measurement of total IgE

Blood samples were collected from the retro-orbital plexus of mice at the end of the experiment. Serum was obtained by centrifugation at 14000 rpm for 5 min and stored in −80 °C until use. The serum total IgE levels were determined by using the mouse IgE ELISA kit (BD Biosciences, San Diego, CA, USA) according to manufacturer’s manual instructions. The reaction product was measured calorimetrically at 450 nm with a microplate reader (BioTekInstrument, Winooski, VT, U.S.A).

### Measurement of cytokine concentration

Inflammatory cytokines such as interleukin (IL)-1β, IL-13 and tumor necrosis factor-alpha (TNF-α) were measured in serum by using multiplex array kit (Bio-Rad, San Diego, CA, U.S.A.) and run on Luminex technology (Bio-Plex Multiplex Bead array system TM, Bio-Rad Hercules, CA, U.S.A.) according to manufacturer’s instruction. Raw fluorescence data were analyzed by software using a 5-parameter logistic method.

### Determination of total ROS

The level of total ROS production in serum was assessed by measuring the oxidation of 2-4-dichlorodihydrofluorescein diacetate (DCFH-DA) (Abcam, Cambridge, MA, U.S.A) by following manufacturer’s manual instructions. In brief, 50 μL of samples were prepared in the 96-well plate. One hundred μL of 10 μM DCFH-DA was added into each well and the plate was incubated for 30 min in the dark. Fluorescence at 488 nm excitation/525 nm emission was analyzed by using DTX-880 multimode microplate reader (Beckman Coulter Inc., Fullerton, CA, U.S.A).

### Measurement of MDA

The level of MDA, a marker of oxidative stressin serum was measured using thiobarbituric acid reactive substances (TBARS) assay kit (Cell Biolabs, Inc., San Diego, CA, U.S.A). The assay was performed according to manufacturer’s instructions. The reaction product was measured calorimetrically at 532 nm with a microplate reader (Biotek instruments, Winooski, VT, U.S.A).

### Measurement of GPx

GPx activity in serum was measured for H_2_O_2_ scavenging capacity by modified Cayman’s GPx assay kit (Cayman Chemical Co., Ann Arbor, MI U.S.A) according to the instruction of the manufacturer. The oxidation of NADPH to NAD^+^ was measured at the absorbance at 340 nm at least 3 times using automated micro plate reader (Beckman Coulter, Inc., Fullerton, CA, U.S.A) at one-minute interval.

### Experimental outcomes

This study provides the in vivo bathing effect of HMW on immunomodulation and redox balance in DNCB-induced AD- like inflammation in hairless mice.

### Statistical analysis

Data values were expressed as the mean ± standard deviation (S.D). The mean values among groups were analyzed and compared using one-way ANOVA followed by subsequent multiple comparison tests (Tukey) with Prism version 5.0 software packages (Graph Pad Software Inc., U.S.A). Significant differences were considered statistically at *p < 0.05, ** *p* < 0.01 and *** *p* < 0.001.

## Results

### HMW bathing ameliorates DNCB-induced skin severity in hairless mice

**Fig. 2:**
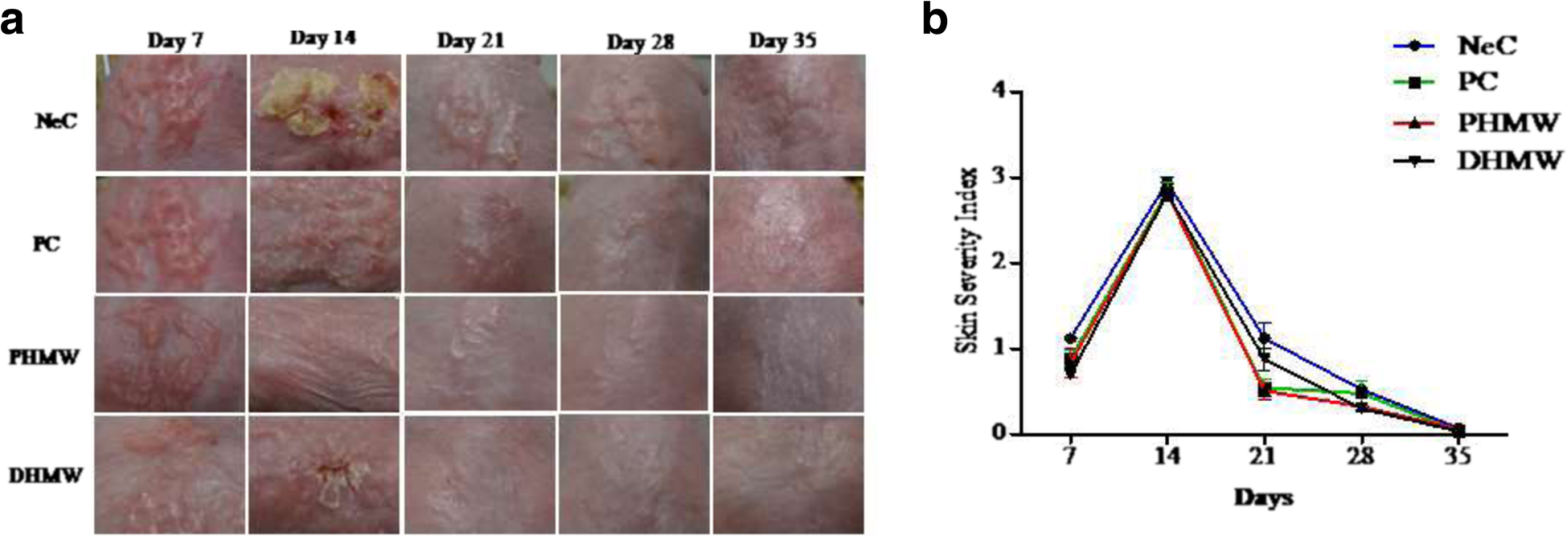
Bathing effects of HMW on clinical skin severity in DNCB -induced hairless mice. **a** The dorsal skin of mice were sensitized by DNCB and treated with experimental materials; photographs of skin lesions from each group of mice were taken every week; **b** The clinical dermatitis scores of DNCB-treated mice were evaluated weekly from 7 to 35 day . Animal groups are represented as Negative control (NeC), Positive control (PC), 100% pure high concentration mineral water (PHMW), 10% diluted high concentration mineral water (DHMW), *n* = 10 respectively

**Fig. 3:**
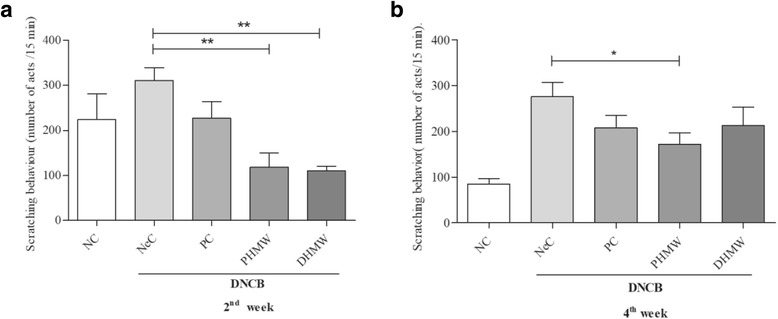
Bathing effects of HMW on scratching behavior during 2nd (**a**) and 4th (**b**) weeks in DNCB-induced hairless mice. Scratching frequency was counted for 15 min in triplicate observation after DNCB-induction in each group. Significant difference was analyzed with ANOVA Tukey’s test, *p < 0.05 and **p < 0.01

To investigate the bathing effects of PHMW and DHMW in DNCB- induced hairless mice, we evaluated skin severity through clinical skin scoring of eczema area, severity index and scratching tendency. The repetitive application of DNCB induced AD-like lesions involving severe skin symptoms in hairless mice. We found that PHMW and DHMW groups showed a slight decrease of DNCB-induced skin severity compared to NeC group (Fig. 2a and b). In the result of scratching behavior test, scratching frequency in PHMW and DHMW was significantly lower than NeC group in the 2nd week of treatment (*p* < 0.01, respectively) (Fig. 3a). In parallel, the scratching frequency in PHMW was significantly decreased compared to NeC group in the 4th week (*p* < 0.05) (Fig. 3b). However, there was no significant difference between eczematous skin lesions.

### HMW bathing decreases serum IgE level in hairless mice with DNCB-induced AD-like inflammation

**Fig. 4:**
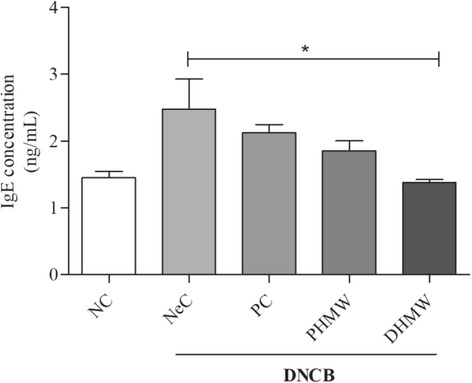
Bathing effects of HMW on serum IgE level in DNCB-induced hairless mice.The level of serum IgE was measured by ELISA**.** Animal groups are represented as Negative control (NeC), Positive control (PC), 100% pure high concentration mineral water (PHMW), 10% diluted high concentration mineral water (DHMW), *n* = 10 respectively. Significant difference was analyzed with ANOVA Tukey’s test, *p < 0.05

AD in hairless mice is known to be frequently mediated by IgE. Thus, to further investigate whether bathing in PHMW and DHMW on DNCB-induced hairless mice, retro-orbital bleeding samples were taken on the last day of the 4th week of treatment. Our data revealed that DHMW group was significantly reduced in serum IgE level compared with NeC group (*p* < 0.05) (Fig. 4). In parallel, the PHMW group also had a lower serum IgE level than the NeC and PC groups although there was not significance (Fig. 4).

### HMW bathing effects on inflammatory cytokines in hairless mice with DNCB-induced AD-like inflammation

**Fig. 5:**
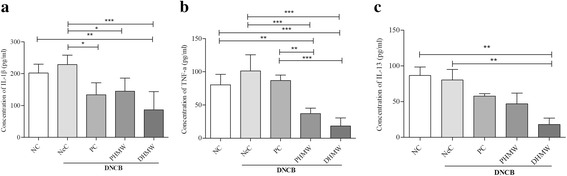
Bathing effects of HMW in serum inflammatory cytokines levels in DNCB- induced hairless mice. The level of serum cytokines was measured with Bioplex Multiplex Bead array system. **a** IL-1β cytokine (**b**) TNF-α cytokine and (**c**) IL-13 cytokine. Animal groups are represented as Negative control (NeC), Positive control (PC), 100% pure high concentration mineral water (PHMW), 10% diluted high concentration mineral water (DHMW), *n* = 10 respectively. Significance difference was analyzed with ANOVA Tukey’s test, *p < 0.05,**p < 0.01 and *** *p* < 0.001

Imbalance of cytokines network has been found in AD. Thus, we examined the effect of PHMW and DHMW bathing on serum cytokine profiles in DNCB-induced hairless mice. We found that IL-1β was significantly inhibited by bathing in PHMW (*p* < 0.05) and DHMW (*p* < 0.001) compared to the NeC group (Fig. 5a). In line, PHMW and DHMW groups showed significantly low level of TNF-α compared to NeC group (*p* < 0.001, respectively) and PC group (*p* < 0.01 and *p* < 0.001) (Fig. 5b). Of note, the level of IL-13 was significantly low in DHMW group compared to NeC group (*p* < 0.01) (Fig. 5c).

### HMW bathing effects on redox balance in hairless mice with DNCB-induced AD-like inflammation

**Fig. 6:**
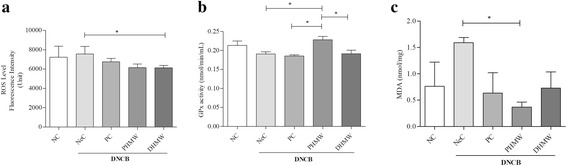
Bathing effects of HMW on serum redox balance markers in DNCB- induced hairless mice. **a** ROS by DCFH-DA (**b**) MDA by thiobarbituric acid reactive substances (TBARS) assay kit (**c**) GPX activity by GPx assay kit. Animal groups are represented as Negative control (NeC), Positive control (PC), 100% pure high concentration mineral water (PHMW), 10% diluted high concentration mineral water (DHMW), *n* = 10 respectively. Significant difference was analyzed with ANOVA Tukey’s test, *p < 0.05

To evaluate the effect of PHMW and DHMW bathing on DNCB induced oxidative stress, we examined the effect of PHMW and DHMW on serum redox marker profiles (ROS, MDA, and GPx) in DNCB-induced hairless mice. We found that, total ROS level was significantly decreased in DHMW group compared to the NeC group (*p* < 0.05) (Fig. 6a). Consistently, serum MDA level was significantly low in PHMW group comapared to NeC group (*p* < 0.05) (Fig. 6b). Additionally, GPx activity was significantly increased in PHMW group compared to NeC, PC and DHMW groups (*p* < 0.05, respectively) (Fig. 6c).

## Discussion

Our study investigated balneotherapeutic effects of HMW on DNCB-induced AD-like inflammation in hairless mice via immunomodulation and redox balance. Several in vivo and clinical investigations have reported the positive therapeutic effect of mineral baths on several skin diseases including AD through its chemical (mineral components) and mechanical effects [21–23]. Cumulative evidence showed that mineral components such as sulphur [12], manganese [13], magnesium [14] and bicarbonates present in spring water exerted beneficial effects on skin disorders such as AD [24]. Studies reported that mineral water rich in sulphur may be absorbed through the skin, exerting beneficial vasodilation, immunomodulatory, anti-inflammatory, keratoplasty, and anti-pruritic effects [25–27]. Inoue et al. reported that balneotherapeutic effect of spring water is useful for controlling skin symptoms of acute exacerbations of refractory cases of AD [13]. In line with these, our study showed HMW (PHMW and DHMW) enriched in high complex minerals (magnesium, calcium, chlorine, manganese, sulphur and strontium ions) was effective against DNCB-induced AD like inflammation in hairless mice (Table 1). Further, this was evidenced in our studies via three ways which are clinicopathological data, immunomodulation and redox balance.

First, we evaluated clinical severity score and scratching frequency of hairless mice. It is well known that AD is often accompanied by clinical symptoms like erythema, edema, excoriations, and dryness along with severe itching which causes scratching. Unexpectedly, bathing in PHMW and DHMW slightly decreased DNCB-induced skin severity (Fig. 2a and b). More importantly, the scratching frequency of PHMW group was significantly lowered than NeC group in both the 2nd and 4th week of bathing (Fig. 3a and b). This might be the first note on balneotherapeutic effects of spring water armed with higher levels of complex minerals against AD-like skin diseases. Next, to secure the immunological clue for clinical relief, we measured serum IgE level in DNCB-induced hairless mice because an elevated IgE level as a hallmark of AD is in proportion to the clinical severity of AD [28, 29]. Consistent with clinical relief, PHMW and DHMW reduced serum IgE levels in DNCB-induced hairless mice when compared with NeC group (Fig. 4). Since IgE is a humoral reflection of Th_2_ immunity, these data might suggest immunomodulation of HMW as a plausible mechanism. In allergic disease, both pro-inflammatory and Th_2_ cytokines play critical roles in the inflammatory manifestation [29, 30]. To further confirm immunomodulation in the host, we measured serum cytokines level in the DNCB-induced hairless mice. Cytokine profiling clearly showed a significant reduction of pro-inflammatory cytokines such as IL-1β, TNF-α, and Th_2_ cytokine level such as IL-13 in HMW bathed mice compared to the NeC mice group (Fig. 5a–c). In AD, epidermal cytokines such as IL-1β and TNF-α act as mediators of inflammatory and immune response. In the sensitization and elicitation phase of allergic dermatitis, IL-1β and TNF-α play a pivotal role [31, 32]. Various chemokines/adhesion molecules which cause the recruitment and proliferating of leukocytes within the skin are produced by TNF-α at the initiation stage of AD. Besides, as cytokine IL-13 is known to be a key stimulator of inflammation and tissue remodeling at sites of Th_2_ inflammation, elevated IL-13 level has been detected in the skin lesions of AD [33, 34]. Taken together, inhibition of pro-inflammatory cytokines including IL-1β andTNF-α as well as reduction of the Th_2_ cytokine IL-13 level might be effective against the overall stages of AD [32, 33]. Consistently, this might be supported by bathing effect with sea water that would ameliorate the AD-like inflammation by way of modulating the production of Th_2_ and pro-inflammatory cytokines in DNCB-induced hairless mice [9].

It is also well known that oxidative stress promotes tissue inflammation through the up regulation of genes that code pro-inflammatory cytokines [35]. Lastly, to explore the linkage of redox imbalance in the pathophysiology of AD, we analyzed different oxidative stress markers. We found that the ROS level of the DHMW group was significantly lower than that of the NeC group (Fig. 6a). Since ROS is considered as one of the important biomarkers of oxidative stress and act as a secondary messenger that can induce the generation of pro-inflammatory and Th_2_ cytokines during inflammatory signaling [35, 36]. Besides this, oxidative stress in AD would be detrimental to lipids, proteins, and DNA. Lipid peroxidation act as an endogenous danger signals that might be responsible for AD pathogenesis, and escalating level of ROS would induce lipid peroxidation [37]. Further, to support this notion, we determined serum MDA level, which is a conventional marker to sense overall lipid peroxidation and oxidative stress. In line, we found significant lower level of MDA in PHMW group as compared to the NeC group (Fig. 6b). Current studies have identified the potential role of lipid peroxidation in numerous pathological condition such as inflammation [37, 38]. Considering the reduction of oxidative effector, ROS, and MDA, our results importantly suggest that bathing with high mineral water is effective against the oxidative stress in DNCB-induced hairless mice. On the other hand, allergic reactions in the skin with allergens is known to affect the antioxidant defense system such as antioxidant enzymes. Thus to examine the bathing effect of HMW on the antioxidant defense system of the DNCB-induced mice, we measured GPx activity in both treatment and control groups and found that there was the significance enhanced activity of GPx in PHMW group compared to the NeC and PC groups (Fig. 6c). This might be partly supported by the balneotherapeutic effect of our high mineral rich spring water increased the activity of GPx activity and is, thereby suggesting a protective role in DNCB-induced AD like inflammation in hairless mice. GPx acts as an important peroxide scavenging enzyme thus offering protection from oxidative stress in tissue by maintaining low levels of ROS [38]. Consistent with our results, several studies have already proven that balneotherapy has potent antioxidant effect in various dermatitis [9, 39, 40]. However, the detailed molecular mechanism underlying the antioxidant effects still remains to be elucidated, and the validated proof in another relevant animal model is required. Given these, our redox profiling showed that HMW might stabilize via enhancing the level of endogenous antioxidants as well as reducing the level of oxidative effectors, thus clinically suggesting its potential beneficial effect against skin diseases including allergic AD-like inflammation. However, further studies with specific analyses of immune-redox makers associated with AD and the relevant signal pathways involved is needed for dissecting the molecular mechanism of balneotherapeutic effect.

## Conclusion

Collectively, our study indicates that bathing with HMW ameliorates DNCB-induced skin inflammation by inhibiting the allergic response (such as serum IgE level and scratching behavior), inflammatory response (such as inflammatory cytokines; IL-1β, TNF-α, and IL-13) in female skh-1 hairless mice. Besides that, levels of redox balance markers (ROS and MDA) were also significantly inhibited with HMW bathing. Taken together, results importantly imply that bathing with HMW might be a safe alternative, a non-medicinal remedy against AD.

## Abbreviations

AD: Atopic dermatitis
ANOVA: One-way analysis of variance
DCFH-DA: 2, 4-dichlorodihydrofluorescediacetate
DHMW: 10% Diluted high mineral spring water
DNCB: 2,4- dinitrochlorobenzene
DW: Distilled water
ELISA: Enzyme –linked immunosorbentassay
GPx: Glutathione peroxidase
HMW: High mineral concentration spring water
IACUC: Institutional animal care and use committee
IgE: Immunoglobulin E
IL: Interleukin
MDA: Malondialdehyde
NADPH: Nicotinamide adenine dinucleotide phosphate
NC: Normal control
NeC: Negative control
PC: Positive control
PHMW: 100% Pure high mineral concentration spring water
ROS: Reactive oxygen species
SA: *Staphylococcus aureus*
TBARS: Thiobarbituric acid reactive substances
TNF-α: Tumor necrosis factor-alpha
